# Retraction: a descriptive study of a manual therapy intervention within a randomised controlled trial for hamstring and lower limb injury prevention

**DOI:** 10.1186/2045-709X-19-24

**Published:** 2011-10-03

**Authors:** Wayne Hoskins, Henry Pollard

**Affiliations:** 1 Department of Chiropractic, Faculty of Science, Macquarie University, NSW 2109, Australia

## Abstract

The journal has been informed by its publisher BioMed Central that contrary to the statement in this article [Wayne Hoskins, Henry Pollard, *Chiropractic & Osteopathy *2010, 18:23], they have been advised by the authors' institution Macquarie University, that its Human Research Ethics Committee did not approve this study. Because the study was conducted without institutional ethics committee approval it has been retracted.

## Retraction

The journal has been informed by its publisher BioMed Central that contrary to the statement in this article [1], they have been advised by the authors' institution Macquarie University, that its Human Research Ethics Committee did not approve this study. Because the study was conducted without institutional ethics committee approval it has been retracted.
